# Tumor necrosis factor-alpha antibody labeled-polyethylene glycol-coated nanoparticles: A mesenchymal stem cells-based drug delivery system in the rat model of cisplatin-induced nephrotoxicity

**DOI:** 10.14202/vetworld.2022.2475-2490

**Published:** 2022-10-29

**Authors:** Faten A. M. Abo-Aziza, Saleh M. Albarrak, Abdel-Kader A. Zaki, Shaymaa E. El-Shafey

**Affiliations:** 1 Department of Parasitology and Animal Diseases, Veterinary Research Institute, National Research Centre, Cairo, Egypt; 2 Department of Veterinary Medicine, College of Agriculture and Veterinary Medicine, Qassim University, Buraydah, Saudi Arabia; 3 Department of Physiology, Faculty of Veterinary Medicine, Cairo University, Giza, Egypt; 4 Department of Physical Chemistry, National Research Centre, Cairo, Egypt

**Keywords:** cisplatin, drug delivery system, infliximab, mesenchymal stem cells, nephrotoxicity, superparamagnetic iron oxide nanoparticles, tumor necrosis factor-alpha

## Abstract

**Background and Aim:**

A delivery system consisting of bone marrow mesenchymal stem cells (MSCs) loaded with polyethylene glycol (PEG) coated superparamagnetic iron oxide nanoparticles (SPIONs) was constructed to treat a rat model of cisplatin (Cis)-induced nephrotoxicity with 1/10 of the common dose of anti-tumor necrosis factor-alpha (TNF-α) antibodies (infliximab).

**Materials and Methods:**

Morphology, size, crystallinity, molecular structure, and magnetic properties of uncoated and PEG-coated SPIONs were analyzed. A delivery system consisting of MSCs containing infliximab-labeled PEG-coated SPIONs (Infliximab-PEG-SPIONs-MSCs) was generated and optimized before treatment. Fifty female Wistar rats were divided into five equal groups: Group 1: Untreated control; Group 2 (Cis): Rats were administered Cis through intraperitoneal (i.p.) injection (8 mg/kg) once a week for 4 weeks; Group 3 (Infliximab): Rats were injected once with infliximab (5 mg/kg), i.p. 3 days before Cis administration; Group 4 (Cis + MSCs): Rats were injected with Cis followed by an injection of 2 × 10^6^ MSCs into the tail vein twice at a 1-week interval; and Group 5 (Cis + Infliximab (500 mg/kg)-PEG-SPIONs-MSCs): Rats were injected with the delivery system into the tail vein twice at a 1-week interval. Besides histological examination of the kidney, the Doppler ultrasound scanner was used to scan the kidney with the Gray-color-spectral mode.

**Results:**

*In vivo*, intra-renal iron uptake indicates the traffic of the delivery system from venous blood to renal tissues. Cis-induced nephrotoxicity resulted in a significant increase in TNF-α and malondialdehyde (MDA) (p < 0.05), bilirubin, creatinine, and uric acid (p < 0.01) levels compared with the untreated control group. The different treatments used in this study resulted in the amelioration of some renal parameters. However, TNF-α levels significantly decreased in Cis + Infliximab and Cis + MSCs (p < 0.05) groups. The serum levels of MDA significantly decreased in Cis + Infliximab (p < 0.05), Cis + MSCs (p < 0.05), and Cis + Infliximab-PEG-SPIONs-MSCs (p < 0.01). Furthermore, the serum activities of antioxidant enzymes were significantly elevated in the Cis + MSCs and Cis + Infliximab-PEG-SPIONs-MSCs groups (p < 0.05) compared to the Cis-induced nephrotoxicity rat model.

**Conclusion:**

With the support of the constructed MSCs-SPIONs infliximab delivery system, it will be possible to track and monitor cell homing after therapeutic application. This infliximab-loading system may help overcome some challenges regarding drug delivery to the target organ, optimize therapeutics’ efficacy, and reduce the dose. The outcomes of the current study provide a better understanding of the potential of combining MSCs and antibodies-linked nanoparticles for the treatment of nephrotoxicity. However, further investigation is recommended using different types of other drugs. For new approaches development, we should evaluate whether existing toxicity analysis and risk evaluation strategies are reliable and enough for the variety and complexity of nanoparticles.

## Introduction

The kidney is a major target organ for the toxic effects of various chemical agents, and thus nephrotoxicity is a frequent object in clinical medicine. Since its authorization for medical use over 40 years ago, the nephrotoxicity of cisplatin (Cis) is well known [1]. As of right now, there is no effective pharmacological treatment to prevent or cure Cis-induced nephrotoxicity [2]. Due to their efficacy and safety, mesenchymal stem cells (MSCs) may provide promising cell therapy for nephrotoxicity [3]. The utilization of stem cells as a therapeutic tool for the treatment of toxicity and inflammation in different tissues has been gaining a growing interest [4]. The renewing of damaged tissues by stem cells is based on their natural capability to migrate to inflammatory sites and generate tissue-specific progenies that directly remodel parenchyma by directing differentiation and organ repair, besides the stimulation of endothelial cells for angiogenesis and neovascularization [5]. Recently, innovative drug delivery technologies have been developed to improve the effectiveness of treatments via supporting their biological availability, reducing their degradation, enabling targeting and consequently regulating their release, cellular uptake, and avoiding undesired side effects [6]. MSCs are interesting as delivery agents due to their innate properties besides their production of biological molecules and immune-modulatory activities [7]. Since MSCs have physiologically retained the “smart targeting,” their ability to migrate/home toward damaged tissues can be augmented by several factors extensively explored in other studies.

Due to their exceptional properties in nanotechnology, superparamagnetic iron oxide nanoparticles (SPIONs) have several uses in the medical industry, particularly in imaging, diagnosing, trafficking, therapy, and drug delivery [8]. There is ongoing research focused on minimizing drug concentrations, toxicity, and other side effects while improving SPIONs-based therapeutic efficacy. Superparamagnetic iron oxide nanoparticles are encased within synthetic polymer coatings that have been used for the past two decades. This approach is employed *in vitro* and *in vivo* settings and MSCs achieved similar mechanisms to cells of areas of interest as with drug loading, where cells can engulf the SPIONs [9]. Further research has revealed that SPIONs-loaded MSCs can improve migratory efficiency *in vivo* without affecting cell viability, phenotype, or differentiation potential [10]. Coating the surface of SPIONs with a shell can protect it from aggregation, opsonization, disintegration, and phagocytosis, which extend its duration in the systemic circulation [11]. The coating can expand the distribution of the drug in injured tissues [12]. Drugs loaded in coated SPIONs could be mitogens, growth factors, antibodies, and morphogens [13, 14].

Thus, the study aimed to construct a drug delivery system based on MSCs loaded with SPIONs that were precoated with anti–TNF-α antibody (Infliximab)-labeled polyethylene glycol (PEG) to treat Cis-induced nephrotoxicity rat model.

## Materials and Methods

### Ethical approval

This study followed the National Institutes of Health’s Guide for the Care and Use of Laboratory Animals. The protocol was approved by the Institutional Animal Care and Use Committee of the National Research Centre, Cairo, Egypt (Protocol Number: 19/151). All surgeries were done under sodium pentobarbital anesthesia, and every attempt was made to minimize the pain.

### Study period and location

This study was conducted from November 2021 to May 2022 at National Research Centre, Cairo, Egypt.

### Isolation of rat bone marrow (BM)-MSCs

Healthy albino male rats with 120 g average weight were used to isolate BM-derived all nucleated cells from the femurs [15]. The isolated cells were expanded by culturing at 10 × 10^6^ cell density into 100 mm culture dishes (Greiner Bio-One, Sigma-Aldrich, USA) in an expansion medium consisting of alpha minimum essential medium (α-MEM, Sigma-Aldrich) containing 20% fetal bovine serum (FBS, Sigma-Aldrich, USA), L-glutamine (2 mM, Sigma-Aldrich, USA), 2-mercaptoethanol (55 μM, Sigma-Aldrich, USA) and penicillin/streptomycin (Sigma-Aldrich, USA). Two days of incubation of cells were done in 5% CO_2_ at 37°C, and then the detached cells were discarded by changing the medium, and the adhered cells were maintained for 2–3 weeks. Several passages were done by sub-culturing the colony-forming adhered cells in the expansion medium. The time on reaching approximately 80% confluence was calculated by daily microscopic observation (CKX-53, Olympus inverted microscope, USA) [16]. Later on, BM-MSCs were collected at the 3^rd^ passage (P3) on reaching 80% confluence for characterization and proliferation assays.

### Proliferation capability of BM-MSCs

Dimethylthiazol-diphenyltetrazolium bromide (MTT, Sigma-Aldrich) was dissolved in PBS at 5 mg/mL and named as the stock solution, then added to wells to be incubated at 37°C for 4 h. Acid-isopropanol was added to all wells, and the plates were then read at 570 nm for quantification of formazan [17].

### Viability and phenotypic analysis of BM-MSCs

Rat BM-MSCs were detached, collected, and centrifuged at 300× *g* for 5 min and then resuspended in the expansion medium. The viability of BM-MSCS was evaluated in an aliquot using Trypan blue stain by counting the stained live cells and excluding the unstained dead cells [18]. Total count = number of cells (16 square) × dilution × 10^4^ = count × 2 × 10^5^/1 mL. The viability % = 100 × number of viable cells/numbers of total cells (viable + dead).

Specific cell surface markers were assessed for BM-MSCs phenotypic analysis [19]. After washing twice with phosphate buffer saline (137 mM NaCl, 2.7 mM KCl, 10 mM Na_2_HPO_4_, and 1.8 mM KH_2_PO_4_, Lonza, Germany) containing 1% bovine serum albumin (Sigma-Aldrich), 0.2 × 10^6^ cells were stained with anti-CD34, anti-CD14, anti-CD90 and anti-CD73 antibodies (BD Biosciences, USA). Labeled cells and determination of positive and negative population percentages were done using a Fluorescence-activated cell sorting (FACS Calibur flow cytometer, BD Biosciences). A negative sample of untreated isotype was used as a control.

### Bone marrow mesenchymal stem cells population doubling time (PDT)

Final PD was calculated by seeding the cells with complete media and using the formula log2 (number of harvested cells/initial number of plated cells) [20]. PDT was calculated as the ratio of incubation period divided by the number of cell doublings at each passage [21].

### Adipogenic differentiation

MSCs were seeded in Dulbecco modified Eagle’s medium (DMEM, 4.5 g/L glucose, Sigma-Aldrich) with 1 μM DEX, 0.5 mM indomethacin, 0.5 mM 3-isobutyl-1- methylxanthine, 10% FBS, 1% (v/v) P/S, 10 μg/mL insulin (Sigma-Aldrich) for 3 weeks. Adipogenesis was evaluated by Oil Red O staining. A stock solution was prepared from Oil Red O (0.5%) and isopropanol (Sigma-Aldrich). To prepare the working solution, 6 mL of the stock solution was mixed with 4 mL of distilled water and kept for 1 h at room temperature (23°C). The cells were fixed with 4% paraformaldehyde (Sigma-Aldrich) for 20 min. Fixed cells were stained with the working solution for 20 min, and then rinsed with PBS. Stained cells were incubated with absolute isopropanol for 15 min, and optical density (OD) was determined at 520 nm [22].

### Osteogenic differentiation

MSCs were cultured for 2 weeks in α-MEM with 20% FBS, 2 mM L-glutamine, 55 μM 2-ME, 100 μM L-ascorbic acid 2-phosphate, 2 mM β-glycerophosphate, 10 nM Dexamethasone, and 100 U/mL penicillin/streptomycin (Sigma-Aldrich) [23]. To confirm osteogenic differentiation, alizarin red staining and alkaline phosphatase (ALP) activity were evaluated. The cells were fixed in 60% (v/v) isopropanol (Sigma-Aldrich) for 1 min then stained with alizarin red, pH 4.1 (Sigma-Aldrich) after rehydration using distilled water. The cells were lysed with 0.1% Triton X-100 (Sigma-Aldrich) for 5 min at room temperature. The ALP activity was assayed in cell lysates using a commercial kit (Thermo Fisher Scientific Inc., USA), and the absorbance was measured at 405 nm [23].

### Synthesis of SPIONs

Chemicals of analytical reagent grade were used without additional purification. The chemical coprecipitation method was used for the preparation of SPIONs [24]. In this method, ferric chloride hexahydrate (FeCl_3_.6H_2_O 99%) and ferrous sulfate heptahydrate (FeSO_4_.7H_2_O, ≥ 99%, Merck, USA) solutions were mixed stoichiometrically with a molar ratio (2:1) under N_2_ vapor gas. NaOH solution (1M) was injected into the mixture drop by drop, and the reaction was sustained under vigorous stirring at room temperature using a magnetic stirrer for 1 h until the pH reached 11. The addition of NaOH (Merck) solution at a rate of 3.0 mL/min was justified in order to achieve uniform precipitation. The SPIONs were formed when the color of the solution changed to dark black. The obtained particles were separated magnetically and then repetitively washed with deionized water and ethanol until the pH was 7. The products were subjected to vacuum dryness at 80°C for 6 h to be characterized. The possible reaction for the formation of Fe_3_O_4_ particles: Fe^2+^ + 2Fe^3+^ + 8NaOH = Fe_3_O_4_↓ + 8Na+4H_2_O.

### Synthesis of polymer coated SPIONs

PEG 6000, (Sigma-Aldrich) coated SPIONs samples were prepared using *ex situ* coating technicality. Here, PEG 6000 (Sigma-Aldrich) was used as a dispersing agent to prevent the aggregation of nanoparticles [25]. Briefly, different loading concentrations (25, 50, 100, 500, 1000, 1500, 2000, and 2500 mg/L) of PEG 6000 dissolved in 10 mL water were sonicated with 0.2 g of the suspended SPIONs for 20 min. The mixed solution was shacked for 24 h at room temperature. After shaking and agitation, the suspended yield was separated using an external magnet and then washed with water and ethanol. The synthesized PEG functionalized Fe_3_O_4_ (PEG-coated SPIONs) was dried at 80°C for 6 h.

### Characterizations of nanoparticles

The morphology of all investigated uncoated and coated SPIONs was determined by scanning electron microscopy (SEM-JEOL, JXA-840A Electron Probe Micro-Analyzer, Japan). Energy-dispersive X-ray (EDX) spectroscopy was applied on an SEM-field emission microscope (Hitachi S-800, Oxford, USA) to assess the atomic concentration of various elements on the top surface of the considered samples. Confirmation of the nanostructure was performed using a transmission electron microscope (TEM, JEOL, Japan). X-ray diffraction analysis was used to determine the crystalline phases of the magnetite and their coated nanoparticles using a Bruker diffractometer (Bruker D8 advance, Germany). The patterns were run with a CuKa1 target with a second monochromator 40 kV, 40 mA. The molecular structure was studied qualitatively using a Fourier transform infrared spectroscopy (FTIR) (6100 JASCO) in the range of 4000–400 cm^−1^. A vibrating sample magnetometer (VSM), (LakeShore 7400; Chicago, IL, USA) was used to investigate the magnetic properties of the magnetite nanoparticles at 300K.

### Preparation of PEG-coated SPIONs labeled with anti–TNF-α antibodies

A chimeric monoclonal an anti–TNF-α antibody (infliximab lyophilized powder, Sigma-Aldrich) was suspended in PEG-coated SPIONs at 34:1 wt/vol ratio resulting in 5 mg/mL anti–TNF-α antibody [13].

### Loading MSCs with Infliximab-labeled PEG-coated SPIONs and determination of the intracellular uptake

After trypsinization and counting the cells, 2 × 10^5^ cells were added to each well of the 6-wells plate. Cells were seeded in a 6-well plate and incubated with 40 nm PEG-coated SPIONs at the iron concentration of 155,119, 87, 64, and 31 μg/mL, in DMEM culture at 37°C for 48 h [26]. Labeled cells were washed with PBS 3 times to remove excess iron. Cells were centrifuged and 32% nitric acid (HNO_3_) was added to each cell sample and heated to boiling. The amount of iron released in the solution was measured by iron kits (Sigma-Aldrich) [27]. Labeled adherent cells were trypsinized and washed with PBS before use.

### Improving labeling of MSCs with coated SPIONs

Poly-l-lysine (Sigma-Aldrich) was used as a vehicle for iron oxide uptake into cells [28]. Cells were seeded and cultured for 24 h in a culture medium containing 25 μg Fe/mL and 0.75 mg/mL poly-l-lysine.

### Cytotoxicity assessment

The cytotoxicity assessment was determined after 24 h using dimethylthiazol-diphenyltetrazolium bromide (MTT) assay. Dimethylthiazol-diphenyltetrazolium bromide (Sigma-Aldrich) was dissolved in PBS at 5 mg/mL (stock solution) and was added to all wells and incubated at 37°C for 4 h. Acid-isopropanol (Sigma-Aldrich) was mixed to all wells and the plates were read at a wavelength of 570 nm [17].

### Experimental design of nephrotoxicity model

Nephrotoxicity was induced in female Wistar rats weighing 120 g on average. Fifty animals were assigned into five groups, ten rats each (caged separately). In Group 1 (untreated control): Animals were kept without any therapy. Cisplatin (8 mg/kg) was injected intraperitoneally (i.p.) once a week for 4 consecutive weeks [1]. The treatment with infliximab (5 mg/kg, i.p. rout) was done once 3 days before Cis administration [29]. Group 2 (Cis): Rats were injected with Cis followed by injection with 1 mL of normal saline. Group 3 (Infliximab): Rats were injected with infliximab. Group 4 (Cis + MSCs): Rats were injected with Cis followed by an injection of 2 × 10^6^ MSCs into the tail vein twice at a 1-week interval. Group 5 (Cis + Infliximab-labeled PEG-coated SPIONs-loaded MSCs): Rats were injected with 2 × 10^6^ PGE coated SPIONs-loaded MSCs into the tail vein twice at a 1-week interval. The authors, who were properly educated in animal care and handling, followed the standards of the National Centre for the Replacement, Refinement, and Reduction of Animals in Research for humane endpoints. All animal welfare concerns were considered during the experiment, which was conducted under sodium pentobarbital anesthesia to reduce pain and discomfort. Throughout the trial, body weight, temperature, and behavioral changes were tracked on a daily basis to serve as precise endpoint criteria. When any animal met the endpoint criterion, it was immediately euthanized for a humane death.

### Ultrasound examination

The rats were detected by ultrasound after 2 weeks from transplantation to detect the kidney lesions [30]. Doppler ultrasound scanner equipped with liner array 12 MHz transducer will be used to scan kidneys with the Gray-mode, color mode, and spectral mode. The scanning parameters for each region, such as the gain, field of view, and time gain control were independently optimized. The shape, borders, and features of kidney lesions, as well as their location, were observed. The renal blood flow end focuses were quantified using color mode and power flow mode. The peak systolic velocity (PSV), end-diastolic velocity, and resistance index (RI) were measured.

### Homing of injected MSCs

Two methods for assessing tissue homing of MSCs were used in the current study. First, an assay of the *in vivo* intra-renal cellular iron uptake by the kidney tissues was done. Three rats from each group were used for collecting blood samples 20 and 40 min post-treatment. Forty minutes following the treatment, animals were slaughtered and kidney samples were collected. The collected tissues and blood samples were used for measuring iron concentration using commercially available kits (Sigma-Aldrich). The second method was by detection of sex-determining region Y (SRY) in a gene (Table-1) on the Y-chromosome of male donor rats in female recipient rats using the conventional polymerase chain reaction (PCR) [31]. The PCR conditions were by incubation at 94°C for 4 min; 35 cycles of 94°C for 50 s, 60°C for 30 s, and 72°C for 1 min; and a final incubation at 72°C for 10 min. About 2% agarose gel electrophoresis was used to separate PCR products and stained with ethidium bromide.

**Table-1:** Primer used in the study.

Primer	Forward (5′-3′)	Reverse (5′-3′)	Accession number
SRY gene	CATCGAAGGGTTAAAGTGCCA	ATAGTGTGTAGGTTGTTGTCC	NM_012772.1

SRY=Sex-determining region Y

### Histological analysis

Kidney tissues were collected 2 weeks after the last transplantation and weighed promptly. For histologic analysis, the 5-mm thickness of paraffin slides was done and stained with Haemotoxylin and Eosin.

### Measurement of plasma TNF-α

Measurement of rat TNF-α cytokine was assessed in plasma after the 4^th^ week of transplantation using a rat-specific kit (Invitrogen, USA).

### Measurement of tissue antioxidant

The dissected weighted kidney samples were homogenized in PBS (pH 7.4) to yield 20% (w/v) homogenate [32]. Cooling centrifugation was done at 4°C for 10 min at 300× *g*. Malondialdehyde (MDA) was determined in the supernatant to exhibit lipid peroxidation. The supernatant was further diluted with PBS to be 2% dilution for the determination of the activities of glutathione peroxidase (GPx), catalase (CAT), and superoxide dismutase (SOD) using Spectrum kits (BioMerieux Ltd, UK).

### Measurement of creatinine and blood urea nitrogen (BUN)

Creatinine was measured in diluted 1:50 urine and undiluted serum using a commercial kit (Sigma Aldrich, USA) [33]. The creatinine clearance was calculated as follows: Creatinine Clearance = urine creatinine × 24 h urine volume × 1440/serum creatinine.

The BUN level in serum was measured using a kit at 340 nm ultra-violet by a spectrophotometer. The following formula was used to calculate BUN: BUN = OD of sample × concentration of standard × 0.47/OD of standard.

### Measurement of K and Na

Serum K and Na were measured by ELYTE 2 colorimetric method to assess renal functions.

### Statistical analysis

Values of data were illustrated as means ± standard errors. Analysis of variance was performed for statistical analysis followed by Duncan’s Multiple Range Test, with p < 0.05 being considered statistically significant. Statistical analysis was conducted with the SAS program 21 (SAS, USA).

## Results

**Figure-1:**
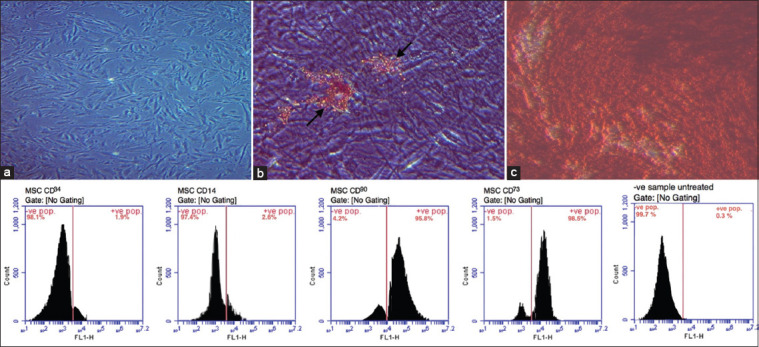
Morphology, immunophenotyping characterization, and differentiation potential of BM-MSCs. (a) Photomicrographically image illustrated the 1ry layer of adhered rat BM-MSCs at 80% confluence. (b) Lipid droplets accumulated by *in vitro* adipogenic differentiated cells as detected by Oil Red O staining (arrow). (c) *In vitro* osteogenic differentiation potential of BM-MSCs as indicated by highly scattered calcified nodules stained red by alizarin red staining (40×). Flow cytometric analyses of BM-MSCs CD markers showed positive stained cells percentages. BM-MSCs negatively expressed CD34 (1.9%) and CD14 (2.6%) markers while positively expressed CD90 (95.8%) and CD73 (98.5%) markers. Isotope was used as −ve untreated control sample (0.3%). Scale bars = 50 μm. BM-MSCs=Bone marrow mesenchymal stem cells.

Cultured MSCs were regularly observed by an inverted microscope until 80% confluence was reached (Figure-1a). Oil Red O staining indicated *in vitro* adipogenic differentiated cells as demonstrated by accumulated lipid droplets (Figure-1b). *In vitro* osteogenic differentiation potential of BM-MSCs was confirmed by highly scattered calcified nodules stained red by alizarin red staining (Figure-1c). Flow cytometric analysis showed the percentages of positively stained cells that indicated the marker of isolated MSCs (Figure-1). Results revealed that MSCs negatively expressed CD34 (1.9%) and CD14 (2.6%) while positively expressed CD90 (95.8%) and CD73 (98.5%).

Serial passages were performed where the viability and cell proliferation were assayed at the 3^rd^ passage. Mesenchymal stem cells reached 80% confluence after 17.438 days and recorded 98.02% viability as indicated by the Trypan blue examination. Formazan OD in confluent cells was 1.224 which indicated cell proliferation. After adipogenic differentiation, the OD of lipid accumulation was 0.636, and the total lipids was 7.503 ug/10 uL. Moreover, OD and concentration of ALP in the osteogenic-differentiated cells were 0.222 and 55.81 U/mg protein, respectively (Table-2).

**Table-2:** Measured parameters of MSCs.

Viability %	Time to reach 80% confluence (days)	Formazan OD (A=570 nm)	Lipid accumulation (OD)	Total lipids (ug/10 uL)	ALP OD (A=405 nm)	ALP (U/mg protein)
98.02 ± 3.71	17.438 ± 3.30	1.224 ± 0.031	0.636 ± 0.094	7.503 ± 1.93	0.222 ± 0.06	55.81 ± 4.91

MSCs=Mesenchymal stem cells, ALP=Alkaline phosphatase, OD=Optical density

### Morphological analysis of SPIONs and PEG-coated SPIONs

**Figure-2:**
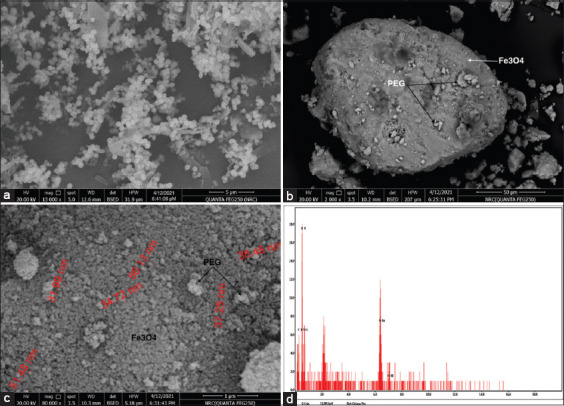
Field emission SEM images and EDX. (a) Iron oxide magnetic nanoparticles at 11,000 × magnification. (b) PEG-coated SPIONs at 2000 × magnification. (c) PEG-coated SPIONs at 80,000 × magnification. (d) EDX of SPIONs for iron oxide magnetic nanoparticles which indicated the presence of Fe and O elements. SEM=Scanning electron microscopy, PEG=Polyethylene glycol, SPIONs=Superparamagnetic iron oxide nanoparticles, EDX=Energy-dispersive X-ray spectrum.

The surface structure of the prepared SPIONs (Figure-2a) and PEG-coated SPIONs (Figures-2b and c) was recorded by SEM. The regular and spherical shapes of SPIONs were shown for all samples. The formation of Fe_3_O_4_ nanoparticles was confirmed by EDX spectra. The Fe was found to be (31.95 wt %) and O (35.85 wt %) which form a Fe-O functional group bond by binding to each other (Figure-2d). Fourier-transform infrared spectroscopy proved the presence of the Fe-O bond.

### Structural analysis of SPIONs and PEG-coated SPIONs

**Figure-3:**
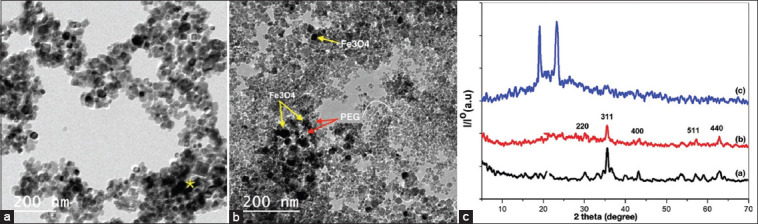
TEM images and XRD patterns. (a) TEM image of SPIONs. (b) PEG-coated SPIONs. (c) XRD patterns of (a) SPIONs, (b) PEG-coated SPIONs, and (c) PEG, 2θ position of magnetite (Fe_3_O_4_) and (PEG) according to ICSD data. TEM=Transmission electron microscope, XRD=X-ray diffraction, SPIONs=Superparamagnetic iron oxide nanoparticles, PEG=Poly ethylene glycol.

The TEM images of both uncoated and coated SPIONs are shown in Figures-3a and b. It was shown that the SPIONs have an average size of 8 and 15 nm with narrow size distribution which is in agreement with the XRD results (Figure-3c). Agglomeration appeared due to the magnetic properties of the prepared SPIONs (Figure-3a). This agglomeration was clearly reduced by adding PEG (Figure-3b). Furthermore, it was seen that the homogeneous shape of SPIONs samples indicated that the PEG acts as a stabilizer and dispersing agent.

According to the obtained XRD picture, all samples have a single-phase spinel cubic structure of SPIONs. This was indicated by appearing all six characteristic peaks belong to the cubic structure of SPIONs at 2θ = 30.1°, 35.4°, 43.1°, 53.2°, 56.9°, and 62.5°, which can be indexed to the (2 2 0), (3 1 1), (4 0 0), (4 2 2), (5 1 1), and (4 4 0) and this matched well with JCPDS (19-0629) card. The existence of the small size of nanoparticles was indicated through those broadening peaks. In the comparison of the two diffraction peaks of the uncoated nanoparticles and that coated with the highest addition of PEG, it can be seen that the diffraction peaks are broader and weaker in intensity for the coated nanoparticles. It indicates that the modification of SPIONs by PEG causes a reduction in crystallinity. Furthermore, no peaks were found for the PEG phase in the sample. This showed that PEG-6000 acted as a template only and did not react or cause any change in the SPIONs phase. The average crystallite size was calculated using the Scherrer equation and an X-ray diffraction pattern [34]: 
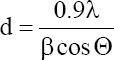
Where 0.9 is the Scherrer constant, l is the X-ray diffraction wavelength (l = 1.5406 Å), b is the full width at half maximum (FWHM) of the plane (311), and q is the Bragg angle in degree [35]. The measured diffraction peak was at a 35.4° angle, the average crystallite size of Fe_3_O_4_ was 16.09 nm, and modified Fe_3_O_4_ nanoparticles were 8.84 nm.

### Chemical analysis of SPIONs and PEG-coated SPIONs

**Figure-4:**
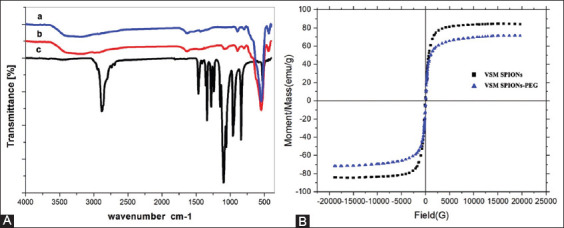
(A) Characterization of surface structure by FTIR and (B) the magnetic properties using VSM. (a) SPIONs, (b) PEG-coated SPIONs, and (c) PEG. FTIR=Fourier transform infrared spectra, VSM=Vibrating sample magnetometry, SPIONs=Superparamagnetic iron oxide nanoparticles, PEG=Poly ethylene glycol.

Fourier-transform infrared spectroscopy characterized the surface chemical structures of PEG-coated SPIONs. Figure-4A (a, b, and c) showed FTIR spectra of SPIONs, PEG-coated SPIONs, and PEG, respectively. The successful preparation of SPIONs and their incorporation in the PEG hybrid were confirmed by the presence of the characteristic peak at 570 cm^−1^ conformable to intrinsic stretching vibrations of the metal at the tetrahedral site (*Fe*tetra↔O), whereas the metal-oxygen band observed at 445 cm^−1^, *v*2 is characteristic to octahedral-metal stretching (*Fe*octa↔O). The affirmations of PEG by the presence of aliphatic C–H stretching and bending vibration of PEG were observed at 2919 and 874 cm^−1^, respectively, in addition to the sharp peak at 1062 cm^−1^ refers to the vibration band of C–O bond in and 1470 cm^−1^ which corresponding to the stretching vibration of C-C group of PEG.

### Magnetic properties of SPIONs and PEG-coated SPIONs

The VSM at 22°C was used to analyze the magnetic properties of both uncoated and coated SPIONs. Figure-4B showed the hysteresis loops of the prepared samples. It can be seen that s-shaped curves appeared over the applied magnetic field, confirming the superparamagnetic behavior of the prepared samples. The magnetization characteristics of the magnetic prepared samples were presented. The saturation magnetization (Ms) was 84.05 and 71.5 emu/g, the remanent magnetization (Mr) was 11.09 and 2.33 emu/g, the squareness (Mr/Ms) was 0.132 and 0.0325, and the coercive field (Hc) was 49.98 and 26.53 Oe for SPIONs and PEG-coated SPIONs, respectively (Table-3).

**Table-3:** Ms, Mr, Mr/Ms, and Hc of SPIONs and PEG-coated SPIONs.

Sample	(*M_s_* emu/g)	(*M_r_* emu/g)	*M_r_*/*M_s_*	*H*_c_ (*Oe*)
VSM SPIONs	84.05	11.09	0.132	49.98
VSM PEG-coated SPIONs	71.5	2.33	0.0325	26.53

Ms=Saturation magnetization, Mr=Remanent magnetization, Mr/Ms=Squareness, Hc=Coercive field, SPIONs=Superparamagnetic iron oxide nanoparticles, PEG=Poly ethylene glycol

### *In vitro* intra-mesenchymal stem cellular iron uptake

Iron concentration in the culture media was determined to verify the *in vitro* intracellular uptake of the infliximab-labeled PEG-coated SPIONs by MSCs (Table-4). After 48 h incubation, the iron concentrations were 109.9, 98.64, 52.69, 35, and 11 mg/dL. By the iron uptake calculations, the results revealed that iron uptake was 45.1, 20.36, 34.31, 29, and 21 mg/dL in infliximab-labeled PEG-coated SPIONs-loaded MSCs achieving 29.1%, 17.1%, 39.4%, 45.3%, and 64.5%, respectively. It was noticed that the higher concentration of infliximab-labeled PEG-coated SPIONs mixture achieved the lowest iron uptake % and vice versa; the lowest concentration recorded the highest iron uptake %. From these results, the ideal concentration of infliximab-labeled PEG-coated SPIONs to achieve the optimum iron uptake by MSCs was 87 mg/dL. The cytotoxicity assessment was determined after 48 h, as evaluated by measuring the OD of the formazan assay in loaded cells. The released quantity of formazan was 1.532 after the incubation with Infliximab-labeled PEG-coated SPIONs at iron concentrations of 87 mg/dL (Table-4).

**Table-4:** *In vitro* intra-mesenchymal stem cellular uptake of Infliximab-labeled PEG-coated SPIONs using iron assay (μg/dL) and OD of formazan assay.

Infliximab-labeled PEG-coated SPIONs mixture	Infliximab-labeled PEG-coated SPIONs-loaded MSCs			Formazan OD

	After 48 h incubation	Iron Uptake	Iron Uptake %	
155	109.9	45.1	29.1	1.526
119	98.64	20.36	17.1	1.577
87	52.69	34.31	39.4	1.532
64	35	29	45.3	1.249
31	11	21	64.5	1.188

SPIONs=Superparamagnetic iron oxide nanoparticles, PEG=Poly ethylene glycol, OD=Optical density

### *In vivo* intra-renal iron uptake

The *in vivo* intra-renal cellular iron uptake in different groups was determined (Table-5). After IV therapy with Infliximab-labeled PEG-coated SPIONs-loaded MSCs, iron concentration significantly elevated in the serum after 20 min and 40 min (p < 0.05) compared to other groups. However, the iron concentration in the serum significantly decreased after 40 min compared to the concentration observed at 20 min post-therapy. Furthermore, the iron concentration in the kidney tissue significantly increased after 40 min of the IV therapy with the infliximab-labeled PEG-coated SPIONs-loaded MSCs (p < 0.05) compared to other groups. These results indicated the *in vivo* intra-renal cellular iron uptake by kidney tissues demonstrating the traffic of infliximab-labeled PEG-coated SPIONs-loaded MSCs from venous blood to renal injuries.

**Table-5:** *In vivo* intra-renal iron uptake in different groups (μg/dL).

Groups/Parameters	Untreated control	Cis	Cis + Infliximab	Cis + MSCs	Cis + Infliximab-labeled PEG-coated SPIONs-loaded MSCs	p-value
Serum iron after 20 min from injection	39.05 ± 1.88	37.48 ± 1.51	41.21 ± 2.77	44.15 ± 2.33	128.55* ± 3.12	0.03121
Serum iron after 40 min from injection	39.17 ± 1.63	40.23 ± 3.52	38.25 ± 3.19	45.20 ± 4.81	83.36* ± 2.17	0.03500
Kidney iron after 40 min from injection	74.22 ± 3.26	66.09 ± 4.54	72.27 ± 4.91	66.73 ± 5.59	112.23* ± 3.51	0.02111

In the same row, *is significantly different at p < 0.05. Cis=Cisplatin; MSC=Mesenchymal stem cells; PEG=Polyethylene glycol; SPIONS=Superparamagnetic iron oxide nanoparticles

### Renal parameters, electrolytes, TNF-α, and antioxidant activity

Cis-induced nephrotoxicity resulted in a significant increase in kidney weight compared to the untreated control group (p < 0.01). Consequently, a disturbance in renal parameters was observed in the Cis-induced nephrotoxicity rat model. Serum BUN, creatinine, and uric acid concentrations were significantly increased (p < 0.01) in the Cis group compared to the untreated control group. However, urine creatinine concentration was significantly decreased (p < 0.05). On electrolyte levels, serum Na and K levels were significantly increased (p < 0.05) in the Cis-induced nephrotoxicity rat model compared to the untreated control. In addition, Cis nephrotoxicity resulted in a significant increase in TNF-α (p < 0.05). The antioxidant MDA was significantly elevated (p < 0.05), while SOD was significantly declined (p < 0.05) in Cis-induced nephrotoxicity rats compared with the untreated control group (Table-5). The different treatments used in this study resulted in the amelioration of some of the examined renal parameters. Briefly, kidney weight and concentrations of BUN, creatinine, and uric acid in the serum significantly declined in the groups treated with Cis + Infliximab, Cis + MSCs and Cis + Infliximab-labeled PEG-coated SPIONs-loaded MSCs compared to the Cis-induced nephrotoxicity rat model (p < 0.05). In contrast, urine creatinine was significantly elevated in Cis + Infliximab compared to the Cis-induced nephrotoxicity rat model (p < 0.05). Moreover, serum Na levels significantly declined in the Cis + Infliximab-labeled PEG-coated SPIONs-loaded MSCs (p < 0.05). However, TNF-α levels significantly decreased in the groups treated with Cis + Infliximab and Cis + MSCs compared to the Cis-induced nephrotoxicity rat model (p < 0.05). Serum level of MDA has significantly decreased in Cis + Infliximab (p < 0.05), Cis + MSCs (p < 0.05) and Cis + Infliximab-labeled PEG-coated SPIONs-loaded MSCs (p < 0.01) compared to Cis-induced nephrotoxicity rat model. However, serum levels of GPx, CAT and SOD were significantly elevated in Cis + MSCs, and Cis + Infliximab-labeled PEG-coated SPIONs-loaded MSCs (p < 0.05) compared to Cis-induced nephrotoxicity rat model (Table-6).

**Table-6:** Measured parameters of rat model of Cis-induced nephrotoxicity and after therapy.

Groups/Parameters	Untreated control	Cis	Cis + Infliximab	Cis + MSCs	Cis + Infliximab-labeled PEG -coated SPIONs-loaded MSCs	p-value
Kidney weight/100 g body weight	0.59 ± 0.08	1.33 ± 0.01**	0.61 ± 0.04^a^	0.63 ± 0.03^a^	0.60 ± 0.07^a^	0.00346
Serum BUN (mg/dL)	92.21 ± 6.49	122.66 ± 5.22*	89.11 ± 3.64^a^	89.43 ± 5.37^a^	90.11 ± 7.74^a^	0.03021
Serum creatinine (mg/dL)	1.01 ± 0.04	1.99 ± 0.01**	1.21 ± 0.04^a^	1.32 ± 0.14^a^	1.38 ± 0.07^b^	0.00216
Uric acid (umol/L)	79.83 ± 3.65	122.16 ± 4.44**	88.65 ± 5.17^b^	94.44 ± 7.54^a^	80.61 ± 3.99^b^	0.0030
Urine creatinine (mg/dL)	31.31 ± 2.77	20.98 ± 2.39*	30.21 ± 3.08^a^	33.54 ± 4.11	32.42 ± 4.81	0.0401
Serum Na (meq/L)	135 ± 7.21	160 ± 4.99*	139 ± 5.43	144 ± 8.34	143 ± 4.93^a^	0.03211
Serum K (meq/L)	3.71 ± 0.66	5.88 ± 0.24*	4.23 ± 0.41	3.74 ± 0.33	4.11 ± 0.56	0.0141
Rat TNF-α (ng/mL)	10.99 ± 1.43	15.72 ± 1.91*	11.44 ± 1.44^a^	10.32 ± 1.04^a^	11.49 ± 3.11	0.0300
MDA (nmol/g tissue)	33.85 ± 3.11	48.22 ± 3.54*	32.43 ± 2.41^a^	35.11 ± 3.76^a^	31.09 ± 3.01^b^	0.0211
GPx (U/g tissue)	65.42 ± 5.39	44.65 ± 4.22	48.33 ± 3.43	69.32 ± 5.61^a^	67.66 ± 5.09^a^	0.0113
CAT (nmol/min/g tissue)	34.60 ± 4.11	27.61 ± 2.43	28.43 ± 3.08	33.72 ± 1.11^a^	36.41 ± 4.87^a^	0.0371
SOD (U/g tissue)	9.11 ± 1.16	5.81 ± 1.31*	6.33 ± 1.40	9.09 ± 1.32^a^	8.76 ± 1.54^a^	0.0151

Values with * and ** are significantly different than untreated control at p < 0.05 and p < 0.01, respectively. Values with a and b are significantly different than Cis group at p < 0.05 and p < 0.01, respectively. Cis=Cis, SOD=Superoxide dismutase, CAT=Catalase, GPx=Glutathione peroxidase, MDA=Malondialdehyde, K=Potassium, Na=Sodium

### Ultrasonography and spectral Doppler

**Figure-5:**
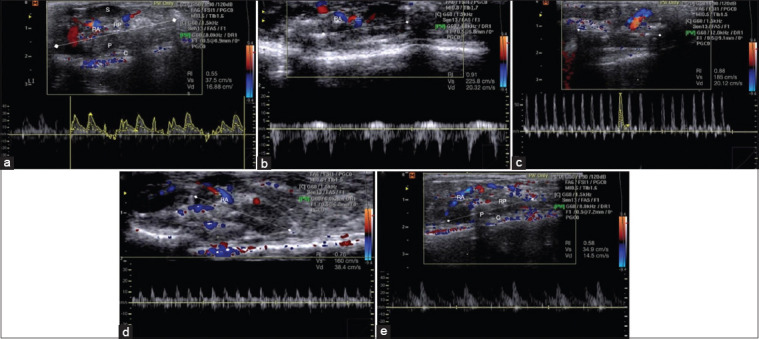
Ultrasonographic appearance of the renal parenchyma and spectral Doppler of renal artery. (a) Normal echogenic kidney with hyperechoic sinus, hypoechoic renal parenchyma showed prominent medullary pyramids and outermost cortex with normal renovascular resistance with PSV of the renal artery was 37.5 cm/s and RI was 0.55. (b) Cis-induced nephrotoxicity group showed glomerulonephritis with increased echogenicity and reduced kidney size and cortical thinning with PSV of the renal artery was 225.8 cm/s and RI was 0.91. (c) Cis + Infliximab group with high echogenicity, a homogeneous architecture with no obvious distinction between the parenchyma and the renal sinus, and a smaller kidney area with high PSV (185 cm/s) and RI (0.88). (d) Cis + marrow mesenchymal stem cells (MSCs) with nearly normal hypoechoic renal parenchyma with medullary pyramids and outermost cortex with PSV (160 cm/s) and slightly high RI (0.76). (e) Cis + Infliximab-labeled PEG-coated SPIONs-loaded MSCs with hypoechoic renal parenchyma, prominent medullary pyramids and outermost cortex with normal PSV (34.9 cm/s) and RI (0.58). S=Sinus, RP=Renal parenchyma, P=Pyramids, C=Cortex, RA=Renal artery, RI=Resistive index, PSV=Peak systolic velocity, EDV=Peak diastolic velocity, SPIONs=Superparamagnetic iron oxide nanoparticles, PEG=Polyethylene glycol. On the US image, the measurement of kidney length is shown by “♦” and a dashed line.

According to an ultrasound scan, the kidney was segmented into the parenchyma and renal sinus. The renal sinus appeared hyperechoic and was formed of the pelvis, fat, and the main intrarenal vessels. The urinary collecting system was not evident in the healthy control group, but it showed a hetereochoic image with the intervening fat and vasculature. The parenchyma was even more hypoechoic and consistent, with the outermost cortex and the innermost, lesser echogenic medullary pyramids (Figure-5a). Higher echogenicity, homogeneous structure with no obvious difference between parenchyma and renal sinus, and decreased renal area were seen in the Cis-induced nephrotoxicity group’s kidneys (Figure-5b). The Cis + Infliximab group’s renal ultrasonography revealed increased echogenicity, homogeneous structure, and no obvious distinction between the parenchyma and the cortex (Figure-5c). However, in the Cis + MSCs group, renal sonography appeared with nearly normal hypoechoic renal parenchyma, medullary pyramids, and outermost cortex (Figure-5d). Moreover, the kidney of Cis + Infliximab-labeled PEG-coated SPIONs-loaded MSCs group indicated hypoechoic renal parenchyma with prominent medullary pyramids and outermost cortex (Figure-5e). Applying spectral Doppler to the renal artery, peak systolic velocities, and resistive index were estimated (Figure-5). Normal PSV of the renal artery (37.5 cm/s) and RI (0.88) were recorded in the healthy control group. However, high PSV (225.8 cm/s) and high RI (0.91) were recorded in the Cis-induced nephrotoxicity group. Furthermore, the Cis + Infliximab group showed high PSV (185 cm/s) and high RI (0.88). Treatment with MSCs recorded PSV (160 cm/s) in the Cis + MSCs group with slightly high RI (0.76). In contrast, Cis + Infliximab-labeled PEG-coated SPIONs-loaded MSCs recoded normal PSV (34.9 cm/s) and RI (0.58).

### Homing of injected MSCs

**Figure-6:**
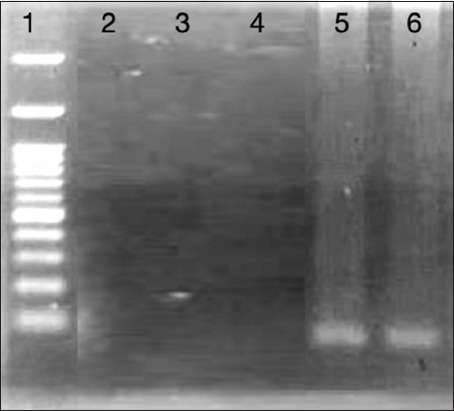
Ultra-violet-transilluminated agarose gel of polymerase chain reaction products of sex-determining region Y gene expression at 104 bp. Lane 1 is polymerase chain reaction marker (100–1000 bp), lane 2 untreated control group, lane 3 Cis induced nephrotoxicity group, lane 4 infliximab treated group, lane 5 Cis + marrow mesenchymal stem cells (MSCs) treated group, lane 6 Cis + Infliximab-labeled polyethylene glycol-coated superparamagnetic iron oxide nanoparticles-loaded MSCs treated group.

By tracking male donor cells in the treated female recipient’s rat’s kidney using conventional PCR, SRY gene of a male donor was detected in the kidney tissue of Cis + MSCs treated and Cis + Infliximab-labeled PEG-coated SPIONs-loaded MSCs treated groups. These findings verified that male donor cells had been found in the injured kidneys of female recipients who had received treatment (Figure-6).

### Histological examination

**Figure-7:**
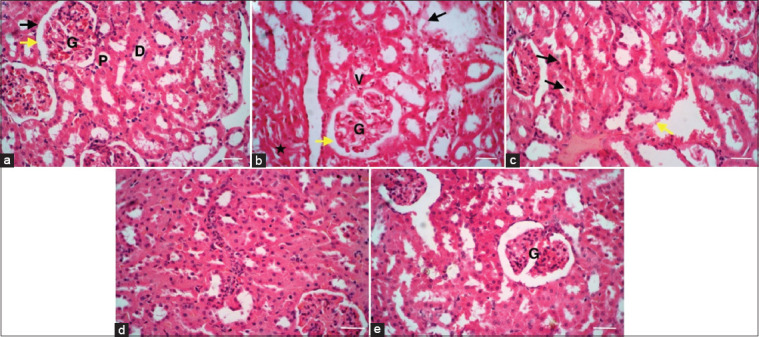
Histological examination of the kidney of different rat groups. (a) The cortex with malpighian corpuscle containing the parietal layer of Bowman’s capsule (black arrow), glomerulus (G), and Bowman’s space were seen in the healthy control group (yellow arrow). Note the proximal (P) and distal (D) tubules are normal. (b) The renal cortex of a Cis-treated rat shows severely shrunk glomeruli (G), a large renal space (yellow arrow), and dilated blood vessels with congested erythrocytes (V). Note the tubular cells with degenerative changes (black star) and necrosis (black arrow). (c) injection of Cis + Infliximab showing renal tubules with necrotic nuclei (black arrow) and necrotic areas (yellow arrow). (d) The kidney showed normal architecture after IV injection of marrow mesenchymal stem cells (MSCs). (e) After IV injection of Cis + Infliximab-labeled polyethylene glycol-coated superparamagnetic iron oxide nanoparticles-loaded MSCs, the glomeruli have open capillary loops and are normocellular (G). There was no evidence of segmentation, necrosis, or sclerosis. There were no histological alterations in the tubular, interstitial, or vascular components, and no evidence of necrosis or inflammation were found (Haemotoxylin and Eosin stain × 40, scale bars = 50 μm).

The cortex with malpighian corpuscle containing the parietal layer of Bowman’s capsule, glomerulus, and Bowman’s space were seen in the healthy control group with normal proximal and distal tubules. The renal cortex of a Cis-treated rat showed severely shrunk glomeruli, large renal space, and dilated blood vessels with congested erythrocytes. The tubular cells with degenerative changes and necrosis were noticed. Injection of Cis + Infliximab resulted in necrotic nuclei and necrotic areas. However, the kidney showed normal architecture after IV injection of marrow mesenchymal stem cells (MSCs). After IV injection of Cis + Infliximab-labeled polyethylene glycol-coated superparamagnetic iron oxide nanoparticles-loaded MSCs, the glomeruli had open capillary loops and were normocellular. There was no evidence of segmentation, necrosis, or sclerosis. There were no histological alterations in the tubular, interstitial, or vascular components, and no evidence of necrosis or inflammation was found (Figure-7).

## Discussion

Researchers have recently been interested in constructing drug delivery systems to enhance targeting and reduce drug doses, but this area is still in its infancy. In the present work, a system of anti–TNF-α antibody (infliximab)-labeled PEG-coated SPIONs-loaded MSCs was developed to treat rat nephrotoxicity induced by Cis.

Combining the properties of MSCs and coated SPIONS for use as drug delivery to diseased kidney tissue is the main goal of the present study. Therefore, the work was planned firstly for the isolation of MSCs from rat BM followed by their characterization. The obtained results showed high cellular viability percentages as measured by Trypan blue and proliferation at the 3^rd^ passage on reaching 80% confluence. Flow cytometric analysis showed 95.8% and 98.5% of CD90 and CD73, respectively, with a negative expression of CD34 (1.9%) and CD14 (2.6%), indicating expression of the common markers of pure and healthy MSCs as previously reported [36, 37]. The total lipids measured after adipogenic differentiation was 7.503 ug/10 uL with the deposited Oil Red O stained lipid droplets that prove adipogenicity as previously obtained [38]. Moreover, the measured concentration of ALP of osteogenic differentiated cells was 55.81 U/mg protein with highly scattered calcified nodules stained red by alizarin red staining. Photomicrograph images were typical of the pictures previously obtained [39]. These mesenchymal images indicate the readiness of the cell for either therapeutic application or labeling as previously reported [40].

The chemical coprecipitation method for the preparation of SPIONs followed by coating with PEG was done as the second step. Six analysis procedures of uncoated or coated SPIONs with PEG were performed by XRD for structural analysis; SEM for shape; EDX for indication of iron; TEM for coating; FTIR for surface structure; and VSM for analyzing the magnetic properties.

XRD indicated that all samples have a single-phase spinel cubic structure of SPIONs as indicated by appearing all six characteristic peaks belong to the cubic structure of SPIONs at 2*θ* = 30.1°, 35.4°, 43.1°, 53.2°, 56.9°, and 62.5°, which can be indexed to the (2 2 0), (3 1 1), (4 0 0), (4 2 2), (5 1 1), and (4 4 0) and this matched well with JCPDS (19-0629) card [41]. The existence of small-size nanoparticles was indicated through those broadening peaks. The comparison of the two diffraction peaks of the uncoated nanoparticles and that coated with the highest addition of PEG revealed that the diffraction peaks are broader and weaker in intensity for the coated nanoparticles. It indicates that the modification of SPIONs by PEG causes a reduction in crystallinity [42]. Furthermore, no peaks were found for the PEG phase in the sample. This showed that PEG-6000 act as a template only and did not react or cause any change in the SPIONs phase [43]. The estimated diffraction peak is at the angle of 35.4° and the average crystallite size of SPIONs is 16.09 nm and modified SPIONs is 8.84 nm.

The properties, physical characteristics, and efficiency of PEG-coated SPIONs changed by functionalization, so they were estimated [44]. The internalization into the cell and lifetime in blood circulation are mostly affected by the morphology and shape of nanoparticles [45, 46].

The SEM examination showed the regular and spherical shape of SPIONs. Among all shapes, the spherical shape of SPIONs is the most suitable for drug delivery applications [47]. The agglomeration due to the magnetic properties of the prepared SPIONs was clearly reduced by adding PEG.

The formation of SPIONs was confirmed by EDX spectra. The Fe was found to be (31.95 wt %) and O (35.85 wt %) which form Fe-O functional group bond by binding to each other. Fourier transform infrared spectroscopy spectroscopy spectra will prove the presence of the Fe-O bond. Fourier transform infrared spectroscopy characterized the surface chemical structures of PEG-coated SPIONs. The successful preparation is indicated by the existence of a distinctive peak at 570 cm^−1^, which corresponds to intrinsic stretching vibrations of the metal at the tetrahedral site (*Fe*tetra↔O), and a metal-oxygen band at 445 cm^1^, v2, which corresponds to octahedral-metal stretching (*Fe*octa↔O) [48]. The affirmations of PEG by the presence of aliphatic C–H stretching and bending vibration of PEG were observed at 2919 and 874 cm^−1^, respectively, in addition to the sharp peak at 1062 cm^−1^ refers to the vibration band of C–O bond and 1470 cm^−1^which corresponding to the stretching vibration of C–C group of PEG [49–52].

VSM at 22°C was used to analyze the magnetic properties of both uncoated and coated SPIONs. Vibrating sample magnetometer showed the hysteresis loops of the prepared samples. It can be seen that s-shaped curves appeared over the applied magnetic field confirming the superparamagnetic behavior of the prepared samples [53]. The TEM image of both uncoated and coated SPIONs showed that the nanoparticles have an average size of 8 and 15 nm with narrow size distribution which agrees with the XRD results. Furthermore, the homogeneous shape of the SPIONs sample was seen, indicating that PEG acts as a stabilizer and dispersing agent [54].

Construction of the infliximab-labeled PEG-coated SPIONs-loaded MSCs delivery system was the third step before treatment. Mesenchymal stem cells were coated with PEG-coated SPIONs, and intracellular uptake was determined, followed by cytotoxicity assessment. Iron concentration in the culture media was determined after 48 h incubation to verify the *in vitro* of the intracellular uptake of infliximab (one over ten the *in vivo* dose 5 mg) (500 μg/two million cells)-labeled PEG-coated SPIONs by MSCs. The results showed that the optimum iron concentration in the solution of infliximab-labeled PEG-coated SPIONs was 87 μg/dL. The cytotoxicity assessment was determined after 48 h of incubation time, as measured by the OD of the formazan in loaded cells. Results revealed that the quantity of release was 1.532 after incubation with Infliximab-labeled PEG-coated SPIONs with the optimal iron concentration of 87 μg/dL. This value expressed by OD of formazan assay in confluent cells was 1.508 which was similar to before coated.

The *in vivo* intra-renal cellular iron uptake in different groups was determined. In the Cis group, iron concentration was significantly increased in kidney tissue than in the control group. Iron accumulation after nephritis was used in another study [55] that referred to the accumulation cause of the renal injury and execration of iron-binding protein. Iron concentration in kidney tissue was significantly increased after 40 min from IV therapy with Infliximab-labeled PEG-coated SPIONs-loaded MSCs compared to other groups. These results indicated *in vivo* intra-renal cellular iron uptake by kidney tissue, indicating the traffic of infliximab-labeled PEG-coated SPIONs-loaded MSCs from venous blood to injured kidney. Measurement of iron in kidney tissue calorimetrically is an easy and cheap method for assessing the traffic of SPIONs-loaded MSCs than other methods [26].

Cis-induced nephrotoxicity resulted in a significant increase in kidney weight compared to the untreated control group. Consequently, a disturbance in renal parameters was observed in the Cis-induced nephrotoxicity rat model. Serum BUN, creatinine, and uric acid concentrations were significantly increased in the Cis group compared to the untreated control group. However, urine creatinine concentration was significantly decreased. On electrolytes levels, serum Na and K levels were significantly increased in the Cis-induced nephrotoxicity rat model compared to the untreated control. The pathological profile, weight, and biochemical parameters of kidneys were discussed and detailed by Nematbakhsh *et al*. [56].

Concerning the immune and oxidative effect of Cis on the kidney, TNF-α was significantly increased as a result of Cis nephrotoxicity, as previously recorded by Jiang *et al*. [57]. Therefore, due to neutralizing effect of infliximab as an antibody against TNF-α, it was used in the present study but with a very low concentration (one over ten). In addition, the antioxidant MDA was elevated and SOD declined in Cis-treated rats compared with the untreated control group. The oxidative load and antioxidant impairment of the kidney by Cis was measured in the previous studies [58, 59] and showed elevation in MDA and lowering in SOD, GSH, and CAT.

Kidney weight and concentration of BUN, creatinine, and uric acid in serum significantly declined in the groups treated with Cis + Infliximab compared to the Cis-induced nephrotoxicity rat model. In addition, TNF-α levels significantly decreased in the groups treated with Cis + Infliximab. Serum levels of MDA significantly increased in Cis + Infliximab treated groups. However, serum levels of GPx, CAT, and SOD were not significantly elevated in the Cis + Infliximab treated group compared to the Cis-induced nephrotoxicity rat model. It is well known that infliximab is a monoclonal antibody against TNF-α and when injected into rat models or even humans with nephrotoxicity, it resulted in amelioration of some immune parameters, kidney biochemical profile, and oxidant-antioxidant system [60]. It directly reacts with TNF-α to inhibit the inflammatory response [61]. Infliximab has different side effects by repeated intravenous administration or the higher dose used in rats such as exposure to infectious and autoimmune reactions [62, 63]. Therefore, in the present study, the main goal is to answer how to deliver the drug to the kidney at a low dose to avoid immune complications and systemic exposure.

Treatment with MSCs used in this study resulted in the amelioration of some renal parameters. Briefly, kidney weight and concentration of TNF-α, BUN, creatinine, and uric acid in serum were significantly declined in the groups treated with Cis + MSCs. Serum level of MDA was significantly decreased, while serum levels of GPx, CAT, and SOD were significantly elevated. The beneficial effect of MSCs to recover nephrotoxicity was discussed by following studies; MSCs’ ability to migrate to the sites of inflammation makes them suitable to be utilized as a part of the delivery system [64]. The mechanism for recovery was through various ways, including anti-inflammation [65], anti-apoptosis, anti-fibrosis, immunomodulation including natural killer cells [66], immune suppression [67], suppression of the expression of some inflammatory cytokines specially TNF-α [68], and proangiogenesis [69]. From the above results, it can be concluded that MSCs have been shown to ameliorate Cis-induced acute kidney injury [70].

Groups treated with Cis + Infliximab-labeled PEG-coated SPIONs-loaded MSCs showed kidney weights and concentrations of TNF-α, Na, BUN, creatinine, and uric acid in serum were significantly declined compared to the Cis-induced nephrotoxicity rat model. The serum levels of MDA were significantly decreased. However, serum levels of GPx, CAT, and SOD were significantly elevated in Cis + Infliximab-labeled PEG-coated SPIONs-loaded MSCs group compared to the Cis-induced nephrotoxicity rat model.

Due to their intrinsic physicochemical features, nanoparticles’ tiny size promotes cellular uptake and can optimize intracellular routes, increasing drug delivery to target regions, as previously discussed [71, 72]. However, this property is not enough to target and treat inflamed tissues [73], and the present study on infliximab was performed to improve the targeting ability of SPIONs.

Mesenchymal stem cells mimicking nanoencapsulations encapsulate pharmaceuticals with a variety of components, such as chemotherapeutic agents, nucleic acids, and proteins [74], and MSCs mimicking nanoencapsulation employ the cell membrane fraction as the capsule and targeting molecules, such as chemokine receptors [75, 76]. The capacity of MSCs to be loaded with infliximab to be released at the site of nephrotic inflammation makes it a superior choice among drug delivery systems. The beneficial effect of this technique was used to minimize the side effects and toxicity of infliximab and improves experimental outcomes [77].

The use of SPIONs in the present study was used due to their biocompatibility and tailor-made surface coatings [78]. SPIONs was also used as an enhancer of contrast between tissues [26]. Furthermore, SPIONs were used to label MSCs isolated from BM with successful tracking by MRI images taken over some time [79]. In addition, MSCs loaded with SPIONs were successfully tracked in the body of a rat [80].

The histological examination of the kidney showed that Cis treated rat showed the renal cortex with highly shrunk glomeruli; wide renal space; and dilatated blood vessels with erythrocytic congestion. The tubular cells showed degenerative changes and necrosis. This picture was like many previous works. Atrophic glomeruli, a dilated urinary space, the lack of proximal convoluted tubule brush boundaries, hypertrophied podocyte pedicles, thicker glomerular basement membrane, and tubular cell vacuolization were all seen [81]. Ozkok and Edelstein [82] mentioned that Cis handling resulted in a decrease in renal blood flow, glomerular filtration rate, ischemia or necrosis of proximal renal tubular epithelial cells with DNA damage, and shedding of the brush shape and apoptosis. Yang *et al*. [83] described the thickening and dilatation of the proximal tubule basement membrane. Injection of Cis + Infliximab showing renal tubules with necrotic nuclei and necrotic areas. The kidney showed normal architecture after IV injection of MSCs. After IV injection of Cis + Infliximab-labeled PEG-coated SPIONs-loaded MSCs, the glomeruli have open capillary loops and are normocellular. There was no evidence of segmentation, necrosis, or sclerosis. There were no histological alterations in the tubular, interstitial, or vascular components, and no evidence of necrosis or inflammation was found. The mechanisms by which MSCs protect the kidney may be by preventing tubular cell apoptosis followed by promoting renal tubular epithelial cell proliferation [84].

Ultrasonographic appearance of the renal parenchyma and spectral Doppler to the renal artery showed normal echogenic kidney with hyperechoic sinus, hypoechoic renal parenchyma showed prominent medullary pyramids and outermost cortex with normal renovascular resistance with PSV of the renal artery was 37.5 cm/s and RI was 0.55. Cis-induced nephrotoxicity group showed glomerulonephritis with increased echogenicity and reduced kidney size and cortical thinning with PSV of the renal artery was 225.8 cm/s and RI was 0.91. Similar pictures were previously obtained [85, 86]. Cis + Infliximab group showed increased echogenicity, a homogeneous structure with a little obvious distinction between the parenchyma and the renal sinus, and a smaller kidney area with high PSV (185 cm/s) and RI (0.88). Cis + MSCs reflect nearly normal hypoechoic renal parenchyma with medullary pyramids and outermost cortex with PSV (160 cm/s) and slightly high RI (0.76). Cis + Infliximab-labeled PEG-coated SPIONs-loaded MSCs exhibited hypoechoic renal parenchyma, prominent medullary pyramids, and outermost cortex with normal PSV (34.9 cm/s) and RI (0.58). In this section of the result, the ultrasonographic technique established here will be beneficial for enhancing kidney disease cell therapy, as recently verified [87]. Furthermore, as compared to other invasive methods, this approach can be built as a clinical application and will be beneficial for cell delivery. As a result, the information obtained from this work can be used to improve cell therapies in a variety of rodent models of renal diseases.

## Conclusion

With the support of the MSCs-SPIONs Infliximab delivery system, it will be possible to easily track and monitor cell homing after therapeutic application. This infliximab-loading system will help the fate of a drug entering the body to the target organ, optimizes the efficacy of therapeutics, and reduces doses. This study’s outcomes contribute to a better understanding of the potential of combining MSCs and nanoparticles with antibodies specific to nephrotoxicity. However, further investigation is recommended using different types of other drugs. For new approaches development, we should evaluate whether existing toxicity analysis and risk evaluation strategies are reliable and enough for the variety and complexity of nanoparticles.

## Authors’ Contributions

FAMA: Designed the study, isolation, proliferation, viability, PDT, and phenotypic analysis of rat BM-MSCs and their adipogenic and osteogenic differentiation. FAMA: Assessed the homing of injected MSCs, histological analysis, and measurement of plasma TNF-α, tissue antioxidant, creatinine, BUN, K, and Na. SEE: Synthesis and polymer coating of SPIONs and their characterizations. AAZ: Labeling with anti-TNF-α antibodies, working with FAMA loading MSCs with infliximab-labeled PEG-coated SPIONs and determination of the intracellular uptake. AAZ and FAMA: Cytotoxicity assessment. AAZ: Nephrotoxicity model and ultrasound examination. AAZ and SMA: Analyzed the data and wrote the manuscript. All authors have read and approved the final manuscript.
